# Analyzing the value of an educational program for psoriasis patients: a prospective controlled pilot study

**DOI:** 10.1186/s12889-019-7778-x

**Published:** 2019-11-19

**Authors:** Corinna Bubak, Marthe-Lisa Schaarschmidt, Lisa Schöben, Wiebke Katharina Peitsch, Astrid Schmieder

**Affiliations:** 1 Department of Dermatology, University Medical Centre Mannheim, Heidelberg University, Theodor-Kutzer-Ufer 1-3, 68135 Mannheim, Germany; 2 Department of Dermatology, Vivantes Klinikum im Friedrichshain, Berlin, Germany

**Keywords:** Psoriasis, Patient education, Educational program, Adherence knowledge about disease, Self-expertise about disease

## Abstract

**Background:**

Psoriasis is a chronic inflammatory skin disease associated with a reduced life-quality. Severe disease forms put the patients at risk for life-treating cardiovascular events, metabolic, and other immune-mediated disorders. Psoriasis patients are often not sufficiently informed about their condition leading to suboptimal treatment adherence and, consequently, worse patient outcome. We investigated the value of an educational program on knowledge and self-expertise about the disease in psoriasis patients in general and dependent on age and disease duration.

**Methods:**

Regular visit psoriasis-patients were asked to participate and choose to receive an additional educational program or not. Participating patients (*n* = 53) filled out two questionnaires: one at study inclusion and one at the next regular visit or after the absolved educational program. Surveys included disease knowledge assessment and numeric rating scales (0–10) for self-expertise about the disease, therapy adherence, and therapy satisfaction. The Dermatology Life Quality Index (DLQI) was used to investigate the quality of life.

All continuous parameters were examined for statistically significant differences by paired t-test or unpaired t-test. Continuous parameters without Gaussian distribution were analyzed with the Wilcoxon matched pairs test or the Mann-Whitney test. For all categorical parameters, Fisher’s exact test was used.

**Results:**

Patients who chose to be educated (*n* = 24) showed a significant increase in knowledge, self-expertise about the disease and amelioration of general health. No positive short-term effects were seen on the quality of life and therapy adherence. Analyzing the effect of age and disease duration, the educational program led to significant improvement of the emotional well-being in older patients (≥50 years) and with a longer disease duration as well as significant amelioration of the self-expertise about psoriasis in younger patients (< 50 years).

**Conclusions:**

Patients who chose to participate in an educational program show a higher gain in knowledge and self-expertise about the psoriatic disease. Educational program thus might have a positive effect on the long-term management of psoriasis. Further long-term studies are needed to provide evidence for the influence educational programs have on outcome, quality of life, and treatment adherence of psoriatic patients.

**Trial registration:**

Deutsches Register Klinischer Studien DRKS00017318 (09.10.2019), retrospectively registered.

## Background

Psoriasis is a chronic inflammatory disease of the skin affecting about 2% of the population in North America and Europe. Different genetic as well as environmental factors such as infections or drugs trigger the onset of the disease leading to a dysregulated immune system and a high production of typical pro-inflammatory cytokines such as tumour necrosis factor (TNF)-α, interleukin (IL)-17, IL-22 and IL-23 [1].

The disease manifests with sharply demarcated, sometimes pruritic and painful, erythematosquamous plaques. Lesions predominantly affect the extensor surfaces of arms and legs, but also involve intertriginous areas, palms, and soles, the scalp or the entire body surface. Severe forms of psoriasis are associated with several comorbidities, including metabolic, cardiovascular, and mental disorders such as anxiety and depression [2]. A causal link for this association is not fully understood, but the systemic inflammatory state and a similar genetic basis may play a part [2]. Treatment options are manifold, including topical agents, phototherapy, traditional systemic therapies, small molecules, and biologicals. A review of treatment options would go beyond the scope of this reading. We kindly refer the reader to excellent reviews in [3, 4].

Because of the stigmatization, pain and skin discomfort caused by the disease, quality of life is markedly reduced – also in patients with mild skin lesions [5, 6]. Only patients with chronic lung diseases and depression have a worse health-related quality of life than psoriasis patients [5, 7]. Besides, psoriasis patients are not as compliant or adherent to therapy regimens as needed [8, 9]. Also, many patients are dissatisfied with their care and lack knowledge about the disease [10, 11].

Hence, an adequate, lifestyle-fitting therapy for the individual patient appears to be crucial. Patient education and self-management-interventions seem to be essential assistance tools for patients to recognize disease aggravating lifestyle factors such as smoking, alcohol consumption and high caloric intake [12, 13], and to start behavioral changes [14]. These tools can help patients increase their general knowledge about the disease and consequently their problem-solving skills, which could lead to higher therapy adherence, quality of life, and a decreased disease severity [14–18]. Scientific evidence for these interventions is still scarce, especially the integrated approach at the tip of each patients’ hand realizing mobile health applications via an application software (Apps).

Here, we present a prospective pilot study carried out to test whether our educational program has a beneficial effect on knowledge and self-expertise about the disease in psoriasis patients in general or dependent on age and disease duration.

## Methods

The study was conducted at the Department of Dermatology, Venereology and Allergology at the University Medical Centre Mannheim, Germany from 09/2016 to 09/2017. The outpatient clinic of this department offers specific consultation hours for psoriasis patients and treats about 500 psoriasis patients per year. Patients attending their regular visits at this outpatient clinic were asked to participate. Inclusion criteria were age ≥ 18 years, physician-confirmed diagnosis of psoriasis (independent of disease-severity and gender), and the ability to provide informed consent. Exclusion criteria were the inability to understand and read German.

Written informed consent was obtained from all patients willing to participate in the study. The informed consent form was available only in German. All patients included in the study were allowed to choose between the intervention group receiving an educational program and the control group, which attended the regular visits every 3 months without an educational program. Patients willingly to participate in the intervention group were offered two educational course dates each consisting of two sessions, where they would receive important background information about their disease.

In total, 65 patients were recruited. Twelfe subjects had to be excluded because they only answered the first questionnaire (*n* = 3, control group) or missed the second educational appointment (*n* = 9, intervention group). In total, 53 participants (35.8% female) were included in the final analyses. Thereof, 24 patients were part of the intervention group and 29 part of the control group.

The study was approved by the Ethics Committees of the Medical Faculty Mannheim, Heidelberg University (Ethics Approval 2016-597 N-MA) and performed according to the principles of the Declaration of Helsinki.

### Data collection

About half of the study population decided to take part in the educational program (intervention group, *n* = 24), which was composed of two 120-min sessions. These participants were asked to fill out a paper-based questionnaire directly after study inclusion and after the second educational session, which took place before the next regular visit. The other half served as control group (*n* = 29). This group completed the same questionnaires as the intervention group immediately after inclusion into the study and about 3 months thereafter during the next regular visit without attending the educational program.

The survey included questions on gender, age, disease duration, weight, height, self-reported body surface area (BSA) (patients were asked to estimate the percentage of the body surface area affected by psoriasis assuming that 1 % of the body surface corresponds to the size of the own palm), comorbidities (psoriatic arthritis, depression, arterial hypertension, cardiovascular disease, hypercholesterolemia, chronic obstructive pulmonary disease (COPD), hepatitis, cirrhosis of the liver or other liver disease, diabetes, cancer, asthma and/or allergies), and questions about smoking. These questions were presented only in the first survey. In both polls, the Dermatology Life Quality Index (DLQI) (range 0 to 30 points) and numeric rating scales (NRS) for therapy adherence, therapy satisfaction, self-expertise about the disease and self-expertise about therapy (range 0 to 10 points) were assessed. Also, participants underwent a short knowledge quiz about psoriasis containing questions on treatment options (2 items), associated comorbidities/addictions (3 items) and pathogenesis (1 item) (range 0 to 35 points). Finally, the survey comprised the Short Form 36 (SF-36) [19]. The SF-36 is a questionnaire that screens the health-related quality of life and consists of nine different items: physical functioning, role limitations due to physical health, role limitations due to emotional problems, energy/fatigue, emotional well-being, social functioning, pain, general health, and health change. Each item is scored on a 0 to 100 scale. A high score indicates a more favorable health state.

### Patient educational program

Two 2-h educational workshops dealing with details on the etiology, pathogenesis, comorbidities, and treatment options in psoriasis in part one and with nutrition, exercise, and addictions in part two were offered. The training was held by two specialists in dermatology (Dr. M. Schaarschmidt and Prof. Dr. A. Schmieder) both senior residents of the Department of Dermatology, Venereology and Allergology at the University Medical Centre Mannheim. All participants of the intervention group were invited to attend the program at the University Medical Centre Mannheim and were given the opportunity to exchange experiences with other patients. At the end of each session, patients were asked to evaluate the workshop with the help of a questionnaire composed of 10 questions about the quality of the lectures, the variety of topics presented, the comprehensibility of contents, the group atmosphere, the personal benefit from the program and the session overall. The score for each question ranged from 1 (“very good”) to 6 (“insufficient”).

### Statistical analysis

All statistical analyses were performed with GraphPad Software (GraphPad Software, Inc., La Jolla, CA 92037 USA). If the D’Agostino and Pearson omnibus normality test showed a Gaussian distribution, all continuous parameters were examined for statistically significant differences by paired t-test or unpaired t-test. Continuous parameters without Gaussian distribution were analyzed with the Wilcoxon matched pairs test or the Mann-Whitney test. For all categorical parameters, Fisher’s exact test was used. Significance was assumed at *p* ≤ 0.05.

## Results

### Study population

The mean age was 46.5 years (range: 19–68 years), the mean disease duration 18.3 years (range: 1–48 years), the mean Body-Mass-Index (BMI) 29.1 kg/m^2^, and the mean Dermatology Life Quality Index (DLQI) 6.4 (range: 0–30). The mean self-reported BSA was only 8.6 as nearly all patients received systemic anti-psoriatic treatment at the time of the study. When asked about their comorbidities, only 14 patients (26.9%) indicated no other diseases in addition to psoriasis. The most frequently reported comorbidity was hypertension (36.5%), followed by psoriatic arthritis (34%), allergies (32.7%), depression (21.3%), and hypercholesterolemia (21.2%). 39% of the study population were current smokers, 31.4% were former smokers, and 29.4% had never smoked before. Baseline characteristics were balanced well between the intervention and control group. All characteristics of the study population are listed in Table 1.

**Table 1:** Characteristics of the study population

Category	No. (%)			*p*-value
	t (*n* = 53)	c (*n* = 29)	i (*n* = 24)	
Gender				
Female n (%)	19 (35.8)	12 (41.4)	7 (29.2)	0,40^a^
Male n (%)	34 (64.2)	17 (58.6)	17 (70.8)	
Age				
Mean (SD)	46.51 (12.0)	46.93 (12.7)	46.0 (11.4)	0,78^b^
Median (25%; 75%)	48.0 (37.5; 56.0)	48.0 (40.5; 57.5)	48.5 (36.5; 54.0)	
Self-reported BSA				
Mean (SD)	8.56 (17.56)	10.25 (20.27)	6.67 (14.1)	0,83^c^
Median (25%; 75%)	2.5 (1.0; 5.0)	2.0 (0.3; 9.0)	2.8 (1.3; 5.0)	
DLQI				
Mean (SD)	6.38 (6.93)	6.28 (7.58)	6.50 (6.22)	0,55^c^
Median (25%; 75%)	4.0 (1.0; 9.0)	4.0 (1.0; 7.5)	5.0 (1.25; 10.0)	
Disease duration				
Mean (SD)	18.13 (12.76)	19.29 (12.38)	16.79 (13.34)	0,49^b^
Median (25%; 75%)	15.0 (9.0; 26.5)	18.0 (9.0; 27.0)	12.0 (7.5; 20.0)	
BMI				
Mean (SD)	29.05 (6.27)	28.32 (6.54)	29.92 (5.95)	0,10^c^
Median (25%; 75%)	27.4 (24.5; 32.1)	25.3 (23.8; 32.4)	28.4 (26.1; 32.5)	
Comorbidity				
Psoriatic arthritis, n (%)	18 (34)	10 (34.5)	8 (33.3)	1,00^a^
Depression, n (%)	11 (21.2)	7 (24.1)	4 (17.4)	0,73^a^
Allergy, n (%)	17 (32.7)	11 (37.9)	6 (26.1)	0,39^a^
Hypertension n (%)	19 (36.5)	10 (34.5)	9 (39.1)	0,78^a^
Cardiovascular diseases, n (%)	3 (5.8)	2 (6.9)	1 (5.5)	1,00^a^
Hypercholesterolemia, n (%)	11 (21.2)	5 (17.2)	6 (26.1)	0,51^a^
COPD, asthma, n (%)	9 (17.3)	7 (24.1)	2 (8.7)	0,27^a^
Hepatitis, cirrhosis of the liver, other liver diseases, n (%)	3 (5.8)	3 (10.3)	0	0,25^a^
Diabetes mellitus, n (%)	5 (9.6)	2 (6.9)	3 (13)	0,64^a^
Cancer, n (%)	1 (1.9)	0	1 (5.5)	0,44^a^
No comorbidities, n (%)	14 (26.9)	8 (27.6)	6 (26.1)	1,00^a^
Current smoker, n (%)	20 (39.2)	11 (39.3)	9 (39.1)	1,00^a^
Ex-smoker, n (%)	16 (31.4)	7 (25)	9 (39.1)	0,37^a^
Non-smoker, n (%)	15 (29.4)	10 (35.7)	5 (21.7)	0,36^a^

^a^Fisher’s exact test; ^b^unpaired t-test; ^c^Mann-Whitney test; *BMI* Body-Mass-Index, *BSA* Body Surface Area (range: 0–100), *c* control, *COPD* Chronic obstructive pulmonary disease, *DLQI* Dermatology Life Quality Index, *i* intervention, *n* number, *SD* standard deviation, *t* total, *25%* 25th percentile, *75%* 75th percentile

### Better knowledge and self-reported expertise in educated patients

**Fig. 1:**
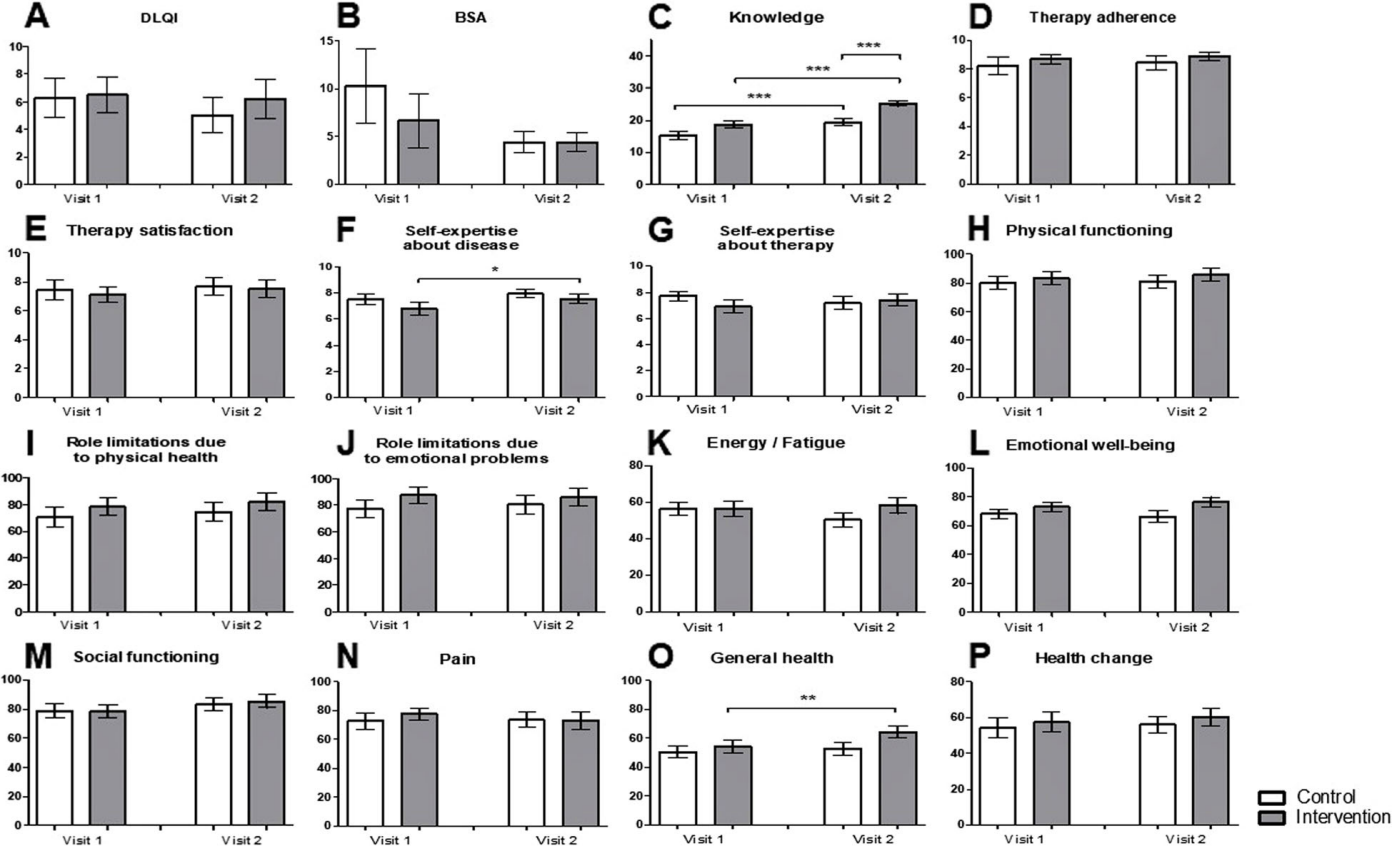
The educational program leads to significant increase in knowledge and self-expertise about psoriasis. **a**-**p** Comparison of the intervention and the control group regarding (**a**) Dermatology Life Quality Index, **b** Body Surface Area, **c** knowledge about psoriasis, **d** therapy adherence, **e** therapy satisfaction, **f** self-expertise about the disease, **g** self-expertise about therapy at visit 1 and visit 2. **h**-**p** Differences between the intervention and control group regarding the (**h**) physical functioning, **i** role limitation due to physical health, **j** role limitation due to emotional problems, **k** energy/fatigue, **l** emotional well-being, **m** social functioning, **n** pain, **o** general health, **p** health change assessed with the Short Form 36 at visit 1 and 2. * *p* ≤ 0.05, ** *p* ≤ 0.01, *** *p* ≤ 0.001. Bars: Means with standard error of the means

Neither the intervention nor the control group reported significant improvement of the self-reported BSA or DLQI between visit 1 and visit 2 (Fig. 1a, b). A statistically significant increase in the general knowledge about psoriasis was noted in both groups between the two visits, although the increment was more pronounced in the intervention group. On average, the control group improved by 4 points in the second questionnaire (*p* = 0.0003; Fig. 1c), whereas the intervention group enhanced their results by about 6.5 points (*p* = < 0.0001; Fig. 1c). The mean score difference between the intervention and the control group on the second visit was 6 points (*p* = 0.0001; Fig. 1c).

Furthermore, patients were asked to indicate their therapy adherence, therapy satisfaction, self-expertise about psoriasis, and self-expertise about anti-psoriatic therapy on a NRS (Fig. 1d-g). A significant amelioration in the self-expertise about the disease between visit 1 and 2 was noted in the intervention group (*p* = 0.0232; Fig. 1f). However, the NRS for this parameter did not differ significantly in the intervention group compared to the control group in visit 2.

In the SF-36 questionnaire, no significant differences regarding physical functioning, physical health, emotional problems, energy/fatigue, emotional well-being, social functioning, pain, and health change were detected between visit 1 and visit 2 of the control and intervention group (Fig. 1h-n and Fig. 1p). However, general health improved significantly by 10% in the intervention group between visit 1 and 2 (*p* = 0.0091; Fig. 1o).

### Significant amelioration of emotional well-being and general health in patients older than 50 years

To evaluate the influence of age on the parameters assessed in the study, patients were divided into two groups with the cut-off set at 50 years. Interestingly, the age group older than 50 years (≥50a) contained a significantly higher percentage of females than the age group younger than 50 years (<50a) (52% versus 21.4%; *p* = 0.0254). As expected, disease duration was longer in the study group aged ≥50 years (mean disease duration: 21.9 versus 14.6 years; *p* = < 0.0001). This finding was associated with a significantly higher prevalence of psoriatic arthritis (48% versus 21.4%; *p* = 0.05) and a significantly lower percentage of patients with no comorbidities (12% versus 40.7%; *p* = 0.03) in the older study population. No significant differences regarding the DLQI, the BMI, and other comorbidities except for psoriatic arthritis were identified, although all comorbidities were more frequent in the subgroup aged ≥50 years. Characteristics of the two age groups are listed in Table 2.

**Table 2:** Characteristics of the subgroups aged < or ≥ 50 years

Category	No. (%)		*p*-value
	<50a (*n* = 28)	≥50a (*n* = 25)	
Group			
Control n (%)	15 (53.6)	14 (56)	1,00^a^
Intervention n (%)	13 (46.4)	11 (44)	
Gender			
Female n (%)	6 (21.4)	13 (52)	**0,0254**^**a**^
Male n (%)	22 (78.6)	12 (48)	
Age			
Mean (SD)	37.71 (9.54)	56.36 (4.31)	***P*** **< 0.0001**^**b**^
Median (25%; 75%)	39.0 (28.5; 45.75)	56.0 (53.0; 58.5)	
Self-reported BSA			
Mean (SD)	10.75 (20.06)	6.10 (14.26)	0,21^c^
Median (25%; 75%)	4.0 (2.0; 9.0)	2.0 (0.275; 5.0)	
DLQI			
Mean (SD)	5.89 (6.01)	6.92 (7.94)	0,73^c^
Median (25%; 75%)	4.0 (1.0; 9.0)	4.0 (2.0; 9.5)	
Disease duration			
Mean (SD)	14.63 (9.68)	21.92 (14.7)	**0,0383**^**b**^
Median (25%; 75%)	14.0 (7.0; 20.0)	19.0 (10.5; 34.0)	
BMI			
Mean (SD)	28.44 (6.34)	29.72 (6.26)	0,31^c^
Median (25%; 75%)	26.45 (24.3; 32.7)	29.0 (25.25; 32.1)	
Comorbidity			
Psoriatic arthritis, n (%)	6 (21.4)	12 (48)	**0,0492**^**a**^
Depression, n (%)	3 (11.1)	8 (32)	0,09^a^
Allergy, n (%)	7 (25.9)	10 (40)	0,38^a^
Hypertension, n (%)	7 (25.9)	12 (48)	0,15^a^
Cardiovascular diseases, n (%)	1 (3.7)	2 (8)	0,60^a^
Hypercholesterolemia, n (%)	4 (14.8)	7 (28)	0,32^a^
COPD, asthma, n (%)	2 (7.4)	7 (28)	0,07^a^
Hepatitis, cirrhosis of the liver, other liver diseases, n (%)	1 (3.7)	2 (8)	0,60^a^
Diabetes mellitus, n (%)	2 (7.4)	3 (12)	0,66^a^
Cancer, n (%)	0	1 (4)	0,48^a^
No comorbidities, n (%)	11 (40.7)	3 (12)	**0,0287**^**a**^
Current smoker, n (%)	9 (32.1)	11 (47.8)	0,39^a^
Ex-smoker, n (%)	8 (28.6)	8 (34.8)	0,76^a^
Non-smoker, n (%)	11 (39.3)	4 (17.4)	0,13^a^

^a^Fisher’s exact test; ^b^unpaired t-test; ^c^Mann-Whitney test; *a* age, *BMI* Body-Mass-Index, *BSA* Body Surface Area (range: 0–100), *COPD* Chronic obstructive pulmonary disease, *DLQI* Dermatology Life Quality Index, *n* number, *SD* standard deviation, *25%* 25th percentile, *75%* 75th percentile

Significant p values (p<0,05) are written in boldface

**Fig. 2:**
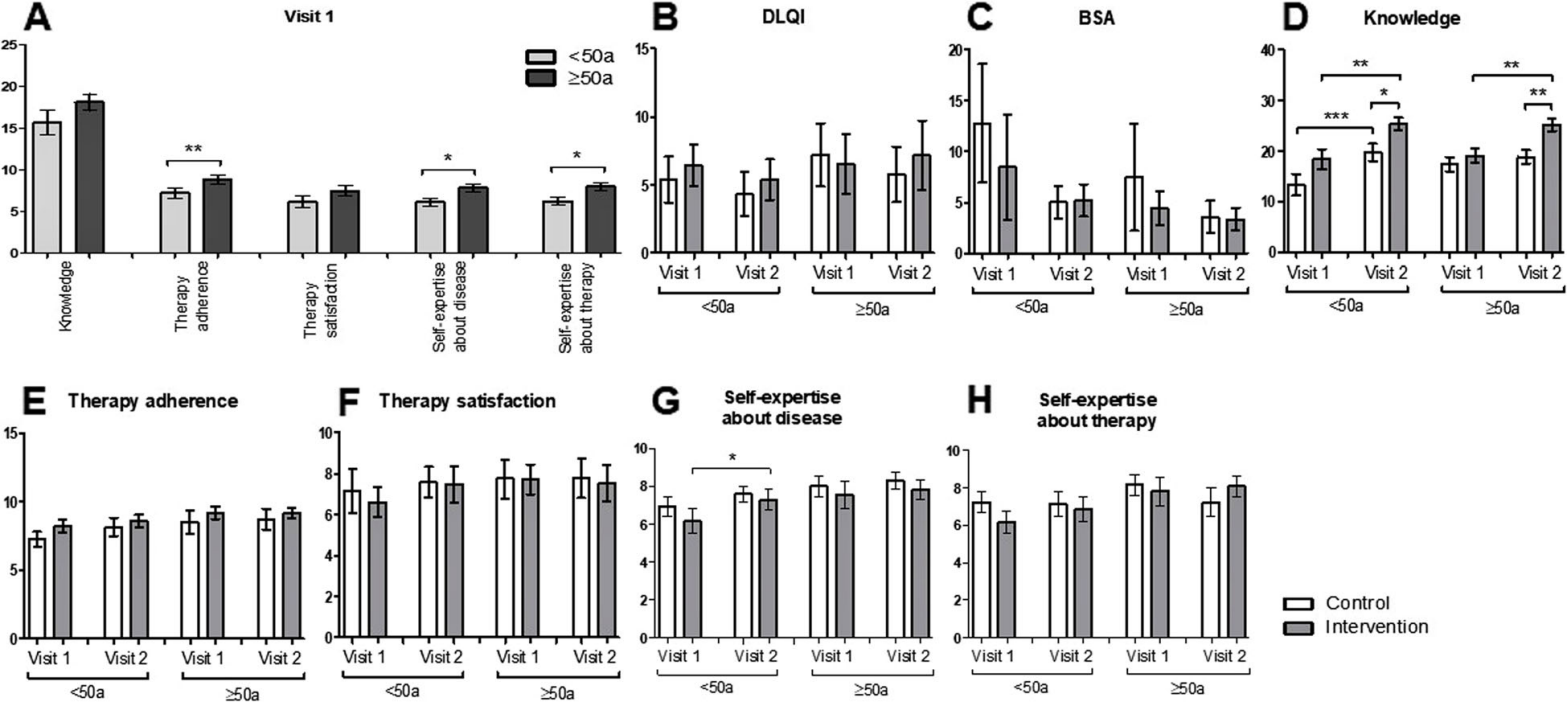
Older patients report higher therapy adherence and self-expertise about the disease and therapy. **a** Differences with regard to knowledge, therapy adherence, therapy satisfaction, self-expertise about the disease and self-expertise about therapy of patients younger or older than 50 years. **b**-**h** Differences between the intervention and control group younger or older than 50 years regarding (**b**) Dermatology Life Quality Index, **c** Body Surface Area, **d** knowledge about psoriasis, **e** therapy adherence, **f** therapy satisfaction, **g** self-expertise about the disease, **h** self-expertise about therapy. * *p* ≤ 0.05, ** *p* ≤ 0.01, *** *p* ≤ 0.001. a: age. Bars: Means with standard error of the means

On visit 1, participants ≥50 years stated a significantly higher adherence to the prescribed treatment compared to younger ones (*p* = 0.0042; Fig. 2a), a higher self-expertise about the disease (*p* = 0.0139; Fig. 2a) and a higher self-expertise about therapy (*p* = 0.0123; Fig. 2a). No difference in the general knowledge about psoriasis, and therapy satisfaction was identified (Fig. 2a).

When further divided into the intervention group (<50a: *n* = 13; ≥50a: *n* = 11) and the control group (<50a: *n* = 15; ≥50a: *n* = 14) no differences in the DLQI and the self-reported BSA was detected. Concerning the general knowledge about psoriasis, both control (*p* = 0.0002) and intervention group (*p* = 0.0035) of the younger study population improved their knowledge between visit 1 and visit 2 (Fig. 2d). The increment was more pronounced in the intervention group when compared on visit 2 (*p* = 0.0153).

In the study group aged ≥50 years only the intervention group showed a significant improvement in the knowledge score between visit 1 and visit 2 (*p* = 0.0014; Fig. 2d). Again, the difference between the control and intervention group at visit 2 was significant (*p* = 0.0029).

**Fig. 3:**
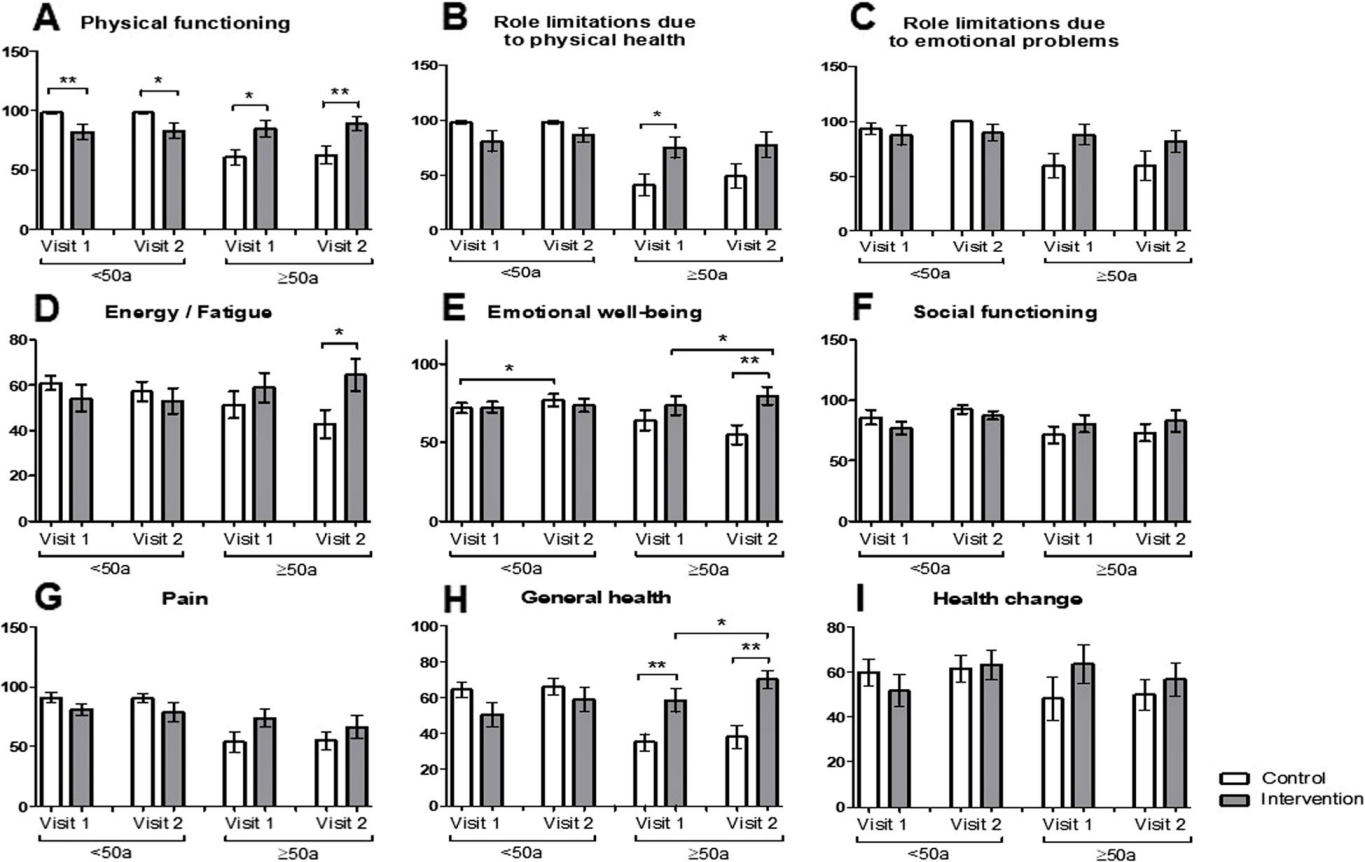
The educational program leads to increase in emotional well-being and general health of older patients. **a**-**i** Comparison of the control and intervention group after stratification into subgroups aged < 50 and ≥ 50 years regarding (**a**) physical functioning, **b** role limitation due to physical health, **c** role limitation due to emotional problems, **d** energy/fatigue, **e** emotional well-being, **f** social functioning, **g** pain, **h** general health, **i** health change assessed with the Short Form 36. * *p* ≤ 0.05, ** *p* ≤ 0.01, *** *p* ≤ 0.001. a: age. Bars: Means with standard error of the means

Concerning therapy adherence, therapy satisfaction, self-expertise about psoriasis and self-expertise about therapy (Fig. 2e-h) significant amelioration in self-expertise about the disease was found in younger members of the intervention group (<50a) (*p* = 0.0218; Fig. 2g). In the SF-36 questionnaire (Fig. 3a-i) significant improvement of the emotional well-being between visit 1 and visit 2 was detected in younger participants of the control group (*p* = 0.0247) as well as in participants ≥50a of the intervention group (*p* = 0.0387) (Fig. 3e). Moreover, an increment in the category “general health” between visit 1 and 2 was identified in participants of the intervention group aged ≥50 years (*p* = 0.0229; Fig. 3h). Significant differences between the control and the intervention group were noticed in the categories “physical functioning”, “role limitations due to physical health”, “energy/fatigue”, “emotional well-being” and “general health” at visit 1 and/or visit 2 (Fig. 3a, b, d, e and h). No significant differences were found in the categories “role limitations due to emotional problems”, “social functioning”, “pain” or “health change” (Fig. 3c, f , g and i).

### The educational program leads to significant amelioration of the emotional well-being in patients with longer disease duration and to significant improvement of the general health and health change in patients with shorter disease duration

To assess the influence of the disease duration on the patients’ profit from the educational program, the study population was divided into patients suffering from psoriasis for more or less than 10 years (dd < 10a versus dd ≥ 10a). As expected, the mean age of the dd ≥ 10a study population was higher than that of the dd < 10a group (49.4 versus 40.8 years; *p* = 0.0148). No significant differences were detected regarding gender, self-reported BSA, DLQI, BMI and comorbidities (Table 3).

**Table 3:** Characteristics of the subgroups with a disease duration < or ≥ 10 years

Category	No. (%)		*p*-value
	dd < 10a (*n* = 17)	dd ≥ 10a (*n* = 35)	
Group			
Control n (%)	8 (47.1)	20 (57.1)	0,56^a^
Intervention n (%)	9 (52.9)	15 (42.9)	
Gender			
Female n (%)	8 (47.1)	11 (31.4)	0,36^a^
Male n (%)	9 (52.9)	24 (68.6)	
Age			
Mean (SD)	40.76 (15.35)	49.37 (9.19)	**0,0148**^**b**^
Median (25%; 75%)	44.0 (24.5; 53.5)	51.0 (44.0; 57.0)	
Self-reported BSA			
Mean (SD)	3.69 (2.7)	10.79 (20.83)	0,79^c^
Median (25%; 75%)	4.0 (2.0; 4.0)	2.0 (0.3; 10.0)	
DLQI			
Mean (SD)	7.82 (6.01)	5.8 (7.39)	0,06^c^
Median (25%; 75%)	6.0 (2.5; 12.5)	3.0 (1.0; 9.0)	
Disease duration			
Mean (SD)	5.59 (2.96)	24.23 (11.1)	***P*** **< 0.0001**^**b**^
Median (25%; 75%)	5.0 (3.5; 9.0)	20.0 (15.0; 31.0)	
BMI			
Mean (SD)	29.19 (7.85)	29.10 (5.53)	0,49^c^
Median (25%; 75%)	26.9 (23.5; 32.5)	27.8 (24.8; 32.8)	
Comorbidity			
Psoriatic arthritis, n (%)	3 (17.6)	15 (42.9)	0,12^a^
Depression, n (%)	2 (11.8)	9 (26.5)	0,30^a^
Allergy, n (%)	5 (29.4)	12 (35.3)	0,76^a^
Hypertension, n (%)	7 (41.2)	11 (32.4)	0,55^a^
Cardiovascular diseases, n (%)	0	3 (8.8)	0,54^a^
Hypercholesterolemia, n (%)	5 (29.4)	6 (17.6)	0,47^a^
COPD, asthma, n (%)	2 (11.8)	7 (20.6)	0,70^a^
Hepatitis, cirrhosis of the liver, other liver diseases, n (%)	0	3 (8.8)	0,54^a^
Diabetes mellitus, n (%)	0	5 (14.7)	0,16^a^
Cancer, n (%)	0	1 (2.9)	1,00^a^
No comorbidities, n (%)	5 (29.4)	9 (26.5)	1,00^a^
Current smoker, n (%)	8 (47.1)	12 (36.4)	0,55^a^
Ex-smoker, n (%)	6 (35.3)	16 (48.5)	0,55^a^
Non-smoker, n (%)	3 (17.6)	11 (33.3)	0,33^a^

^a^Fisher’s exact test; ^b^unpaired t-test; ^c^Mann-Whitney test; *a* age, *BMI* Body-Mass-Index, *BSA* Body Surface Area (range: 0–100), *COPD* Chronic obstructive pulmonary disease, *dd* disease duration, *DLQI* Dermatology Life Quality Index, *n* number, *SD* standard deviation, *25%* 25th percentile, *75%* 75th percentile

Significant p values (p<0,05) are written in boldface

**Fig. 4:**
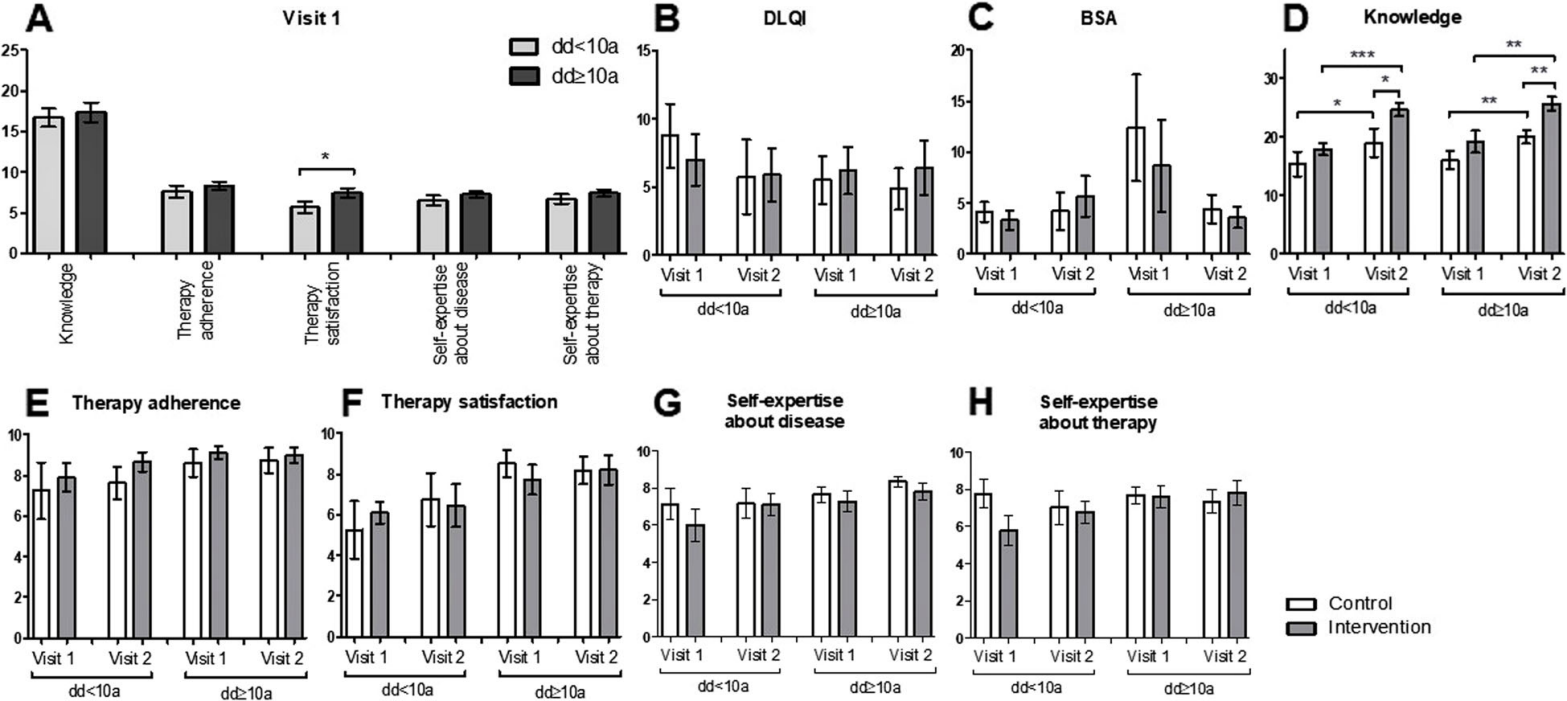
Patients with longer disease duration are more satisfied with their treatment. **a** Differences between patients with a disease duration shorter or longer than 10 years regarding knowledge, therapy adherence, therapy satisfaction, self-expertise about the disease and self-expertise about therapy. **b**-**h** Comparison of the intervention and the control group after stratification according to disease duration (< 10 or ≥ 10 years) in terms of (**b**) Dermatology Life Quality Index, **c** Body Surface Area, **d** knowledge about psoriasis, **e** therapy adherence, **f** therapy satisfaction, **g** self-expertise about the disease, **h** self-expertise about therapy. * *p* ≤ 0.05, ** *p* ≤ 0.01, *** *p* ≤ 0.001. a: age; dd: disease duration. Bars: Means with standard error of the means

On visit 1, patients with a longer disease duration were more satisfied with their therapy than the others (*p* = 0.0165; Fig. 4a). No significant differences were observed in the knowledge test, therapy adherence, self-expertise about the disease and self-expertise about therapy (Fig. 4a).

When further divided into the control (dd < 10a: *n* = 8; dd ≥ 10a: *n* = 20) and the intervention group (dd < 10a: *n* = 9; dd ≥ 10a: *n* = 15), the dd < 10a and the dd ≥ 10a study population showed a significant gain in knowledge about the psoriatic disease in the control (*p* = 0.0221 and *p* = 0.0083; Fig. 4d) as well as intervention group (*p* = 0.0007 and *p* = 0.003; Fig. 4d). Again, the intervention group of both study groups scored on average 5.5 points higher than the control group on visit 2 (*p* = 0.0453 and *p* = 0.0019; Fig. 4d). No differences in the DLQI, the self-reported BSA, therapy adherence, therapy satisfaction, self-expertise about the disease and self-expertise about therapy were detected between visit 1 and visit 2 (Fig. 4b, c, e, f, g and h).

**Fig. 5:**
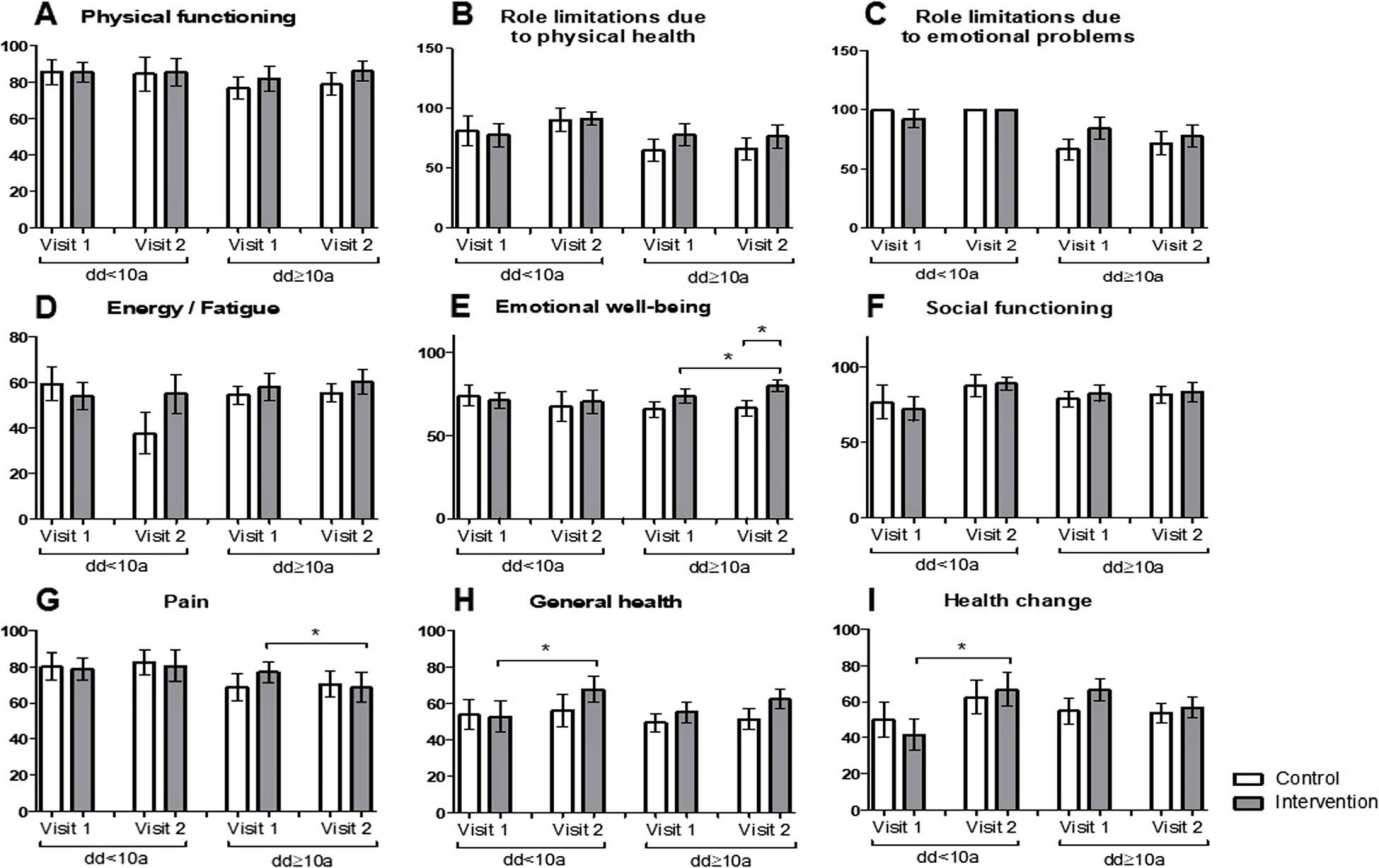
Effects of the educational program on patients with a shorter or longer disease duration. **a**-**i** Differences between the intervention and the control group with a disease duration shorter or longer than 10 years regarding (**a**) physical functioning, **b** role limitation due to physical health, **c** role limitation due to emotional problems, **d** energy/fatigue, **e** emotional well-being, **f** social functioning, **g** pain, **h** general health, **i** health change assessed with the Short Form 36. * *p* ≤ 0.05, ** *p* ≤ 0.01, *** *p* ≤ 0.001. a: age; dd: disease duration. Bars: Means with standard error of the means.

In the SF-36 questionnaire, a significant increment in the emotional well-being of the intervention group with longer disease duration was noticed (*p* = 0.0491; Fig. 5e). The dd ≥ 10a study population with intervention had significantly better emotional well-being than the dd ≥ 10a study population without intervention at visit 2 (*p* = 0.0405; Fig. 5e). In the same study population, the pain worsened between visit 1 and visit 2 in the intervention group (*p* = 0.0496; Fig. 5g). When looking at the dd < 10a study population, significant improvement in general health (*p* = 0.0472; Fig. 5h) as well as in the health change-category (*p* = 0.04; Fig. 5i) was noticed. No differences in physical functioning, role limitations due to physical health, role limitations due to emotional problems, energy/fatigue, and social functioning were identified (Fig. 5a, b, c, d and f).

## Discussion

In this prospective controlled pilot study, it was examined whether psoriasis patients - independent of the severity of the disease - would benefit from an educational program regarding knowledge and self-expertise, as we believe that this leads to higher treatment adherence and thus a more favorable outcome for the patients. Besides, the influence of age and disease duration on these variables was assessed as life experience in general or concerning the illness might per se already has a positive effect on disease knowledge and self-expertise. Furthermore, quality-of-life measures such as the DLQI and the SF-36 as well as treatment satisfaction and adherence scores were part of the study. Based on the data presented here, all educated patients showed a significant increase in knowledge, self-expertise about their disease and general health after attending the educational program. A higher level of knowledge achieved by an educational program for psoriasis patients was also assessed in a study by Nagarajan et al. in which 52 participants received a video-assisted teaching program for 3 months [20]. In this study, knowledge about the disease did not increase in the control group. In our study, increase in knowledge about psoriasis was detected both in the control and in the intervention group. This finding may be explained by the fact that participation in a clinical trial, which is associated with more medical attention, leads to an active confrontation with the disease. This hypothesis is supported by results from a patient survey conducted by a clinical research group (theavocagroup.com) in which patients´ reasons to participate in clinical trials were assessed. 51% of the patients stated that they participate in clinical trials to learn more about their condition (www.theavocagroup.com). In our study, a far higher increment in knowledge was assessed in the intervention group compared to the control group. Further evidence for the effectiveness of educational programs for psoriasis patients comes from a review in which 16 clinical studies were analyzed [17]. Although the strength of evidence was considered low and the long-term effects for patients were not regarded as proven, nearly all reviewed studies reported an increase in the quality of life of educated patients. In contrast, effects on the disease severity were inconsistent [10, 11, 17]. In our study neither improvement in the quality of life nor a better outcome was seen, but the short observation period of this study might be the reason for this negative result. Instead, we detected an amelioration of general health and a higher self-expertise about the disease after the teaching sessions. The amelioration in the category “general health” may come from a higher awareness of educated patients regarding a healthier lifestyle after being confronted with psoriasis-associated comorbidities and their detrimental effects. Also, data presented in the program might have motivated patients to seek more information on the topic leading to a higher self-expertise about their condition. Consistent with these data, a telephone-based motivational interviewing intervention conducted in Spain resulted in a higher self-efficacy and a positive health behavior change in psoriasis patients after 6 months [18, 21].

When stratifying patients in two age groups (<50a and ≥ 50a), there was a significantly higher percentage of women in the elderly group compared to the younger group. In addition, the average disease duration was longer in the older group and comorbidities, such as psoriatic arthritis, were more frequent. The latter two results are not surprising, but the higher percentage of women in the older cohort is worth discussing in more detail. A fall in the hormone levels, especially estrogen, during menopause is believed to result in higher production of IL-12 and TNF-α and thereby to a higher antigen presentation rate of dendritic cells leading to a worsening of psoriasis [22]. This basic immunological data is supported by results from a clinical trial in which 48% of menopausal women reported an exacerbation of the disease, while only 2% showed improvement [23]. Although the level of evidence for the hormonal influence on the psoriatic disease is scarce, the higher percentage of female patients with moderate-to-severe psoriasis after menopause suggests an association between hormone levels and disease severity and might explain the higher rate of females in our older patient group [24]. Another interesting finding was a higher therapy adherence, self-expertise about disease and therapy reported by patients older than 50a at the beginning of the study. These results are in line with a study from Saeki et al. in which older patients show a higher adherence to systemic drugs [25, 26] but these data were not confirmed in a larger systematic review [27]. Although data regarding the influence of age on therapy adherence, self-expertise about the disease and therapy are inconsistent, it seems plausible that older patients with a longer history of psoriasis as assessed in this study acquire more experience with their skin condition and actively decide to adhere more strictly to the prescribed medication in order to avoid flare-ups.

Further sub-analyses of patients <50a and ≥ 50a and with a disease duration <10a and ≥ 10a showed that only patients <50a increased their self-expertise about the disease by the educational program, while only patients older than 50a reported an amelioration of the emotional well-being as well as general health after the intervention. Emotional well-being also increased in the intervention group of patients with a disease duration ≥10a, while in patients with a disease duration <10a the intervention resulted in an improved health level. These results indicate that especially younger patients quickly gain expertise on their disease after an educational program, while older patients seem to profit more on the emotional and health level. Reich et al. assessed patients’ apprehension of psoriasis [25]. They found that especially older patients with longer disease duration had a more realistic view on their psoriasis and therefore, did not expect a rapid cure [25]. These patients could be more prone to accept and profit from recommendations given in the educational program on an emotional and health level, while younger patients might need time to cope with the new and maybe disturbing information about their disease, the associated comorbidities, and the prognosis. However, this hypothesis needs evaluation after a more extended follow-up period.

In general, characteristics of the control and the intervention group were well balanced.

Significant limitations of our study are the monocentric design and the limited generalizability of the results because of the restricted number of participants. It also has to be taken into account that due to the limited patient number, differences between subgroups might have been missed or over-interpreted. Participants were not randomized in this study but could decide by themselves if they wanted to join the educational program or the control group. This decision was often based on the time patients were willing to invest, and on their level of motivation. It is conceivable that patients of the intervention group had better knowledge and expertise about psoriasis because they were more interested in the optimal management of their disease. Also, the BMI and the BSA were self-reported and is, therefore, a subject to bias.

## Conclusions

Overall, our educational program had a positive impact on the patients’ knowledge and expertise about the disease. Delivery of valid, understandable, and reliable medical information is essential to empower and motivate patients for self-management of their disease, to improve treatment adherence, and optimize outcome. Clearly, our findings need to be verified in more extensive prospective trials. As a consequence of the promising results of our pilot study, a prospective randomized clinical study has been initiated at our Medical Centre. This study will include a larger number of participants and comprise a longer monitoring period. In addition to the educational program, study patients will receive a specifically developed psoriasis App to allow more frequent follow-ups. Based on the data presented here, we believe that educational programs for psoriasis patients should be routinely offered at psoriasis centers to improve patient care.

## Data Availability

The datasets used and analyzed during the current study are available from the corresponding author on reasonable request.

## Abbreviations

%: percentage
a: years
app: application software
BMI: Body-Mass-Index
BSA: Body Surface Area
COPD: Chronic obstructive pulmonary disease
dd: disease duration
DLQI: Dermatology Life Quality Index
IL: Interleukin
n: number
SD: Standard deviation
TNF: Tumour necrosis factor
